# Does the Reliability and Accuracy of the Judet and Letournel Classification System for Acetabular Fractures Increase using a Novel Algorithm?

**DOI:** 10.5704/MOJ.2507.008

**Published:** 2025-07

**Authors:** G Jain, R Datt, S Lenka, G Vadivelu, A Krishna, A Mahmood

**Affiliations:** 1 Department of Orthopaedics, All India Institute of Medical Sciences, Bhubaneswar, India; 2 Department of Orthopaedics, ESIC Medical College and Hospital (MCH), Faridabad, India; 3 Department of Orthopaedics, SUM Hospital, Bhubaneswar, India; 4 Department of Orthopaedics, St John's Medical College, Bengaluru, India; 5 Department of Orthopaedic Surgery, Maulana Azad Medical College (MAMC), New Delhi, India; 6 Department of Orthopaedics, All India Institute of Medical Sciences, New Delhi, India

**Keywords:** acetabulum, fracture, classification, algorithm, Judet-Letournel classification system

## Abstract

**Introduction:**

We have devised an algorithm to assist classifying acetabulum fractures using plain radiographs. This study aimed to test if the accuracy and reliability of fracture classification increases using our algorithm in resident doctors.

**Materials and Methods:**

Seventy-two residents of eight tertiary care institutes took part in our survey. These residents were divided into three groups, Groups A, B, and C, with 31, 20, and 21 residents, respectively. Two different Collections (1 and 2) containing radiographs of twenty patients each, with known classification from CT and intra-operative findings, were prepared. Collection 1 radiographs were given to Group A and B, and Collection 2 radiographs were given to Group C. Group A residents were asked to classify the fractures using our algorithm, and Group B and C residents were asked to classify the fractures according to their understanding. Intra-observer and interobserver reliability were estimated.

**Results:**

A total of 1411 unique responses were made. The accuracy of group A, B, and C residents was 53.8%, 34.9% and 28.3%, respectively (p-value 0.001). The interobserver reliability for fracture classification was fair with an algorithm (κ = 0.32) and slight without an algorithm. The intra-observer reliability among five observers was moderate (κ = 0.43).

**Conclusion:**

Our algorithm improves the accuracy and reliability for classifying acetabular fractures according to the Judet-Letournel classification for resident doctors with two to four years of experience.

## Introduction

Acetabular fractures are one of the most challenging fractures to deal with, primarily because of the complex anatomy of the pelvis bone, leading to difficulty in understanding the fracture morphology from pre-operative radiography. The Judet-Letournel classification system continues to be the most critical guide for acetabular surgeons in deciding the surgical approach and overall fracture management^1^. Though, with the advent of 3D CT scans, the understanding regarding fracture classification has dramatically improved^2,3^ interpretation of plain radiography is of utmost importance as intra-operative fluoroscopy has a significant role in fracture reduction and fixation^4^.

Previous literature suggests that young surgeons, particularly resident doctors, have poor interobserver reliability with a kappa value of 0.24-0.27 and only 11% accuracy while classifying acetabular fractures according to Judet-Letournel classification from plain radiographs^5,6^. Nevertheless, the interobserver reliability is moderate when used by musculoskeletal radiologists (κ = 0.42) and substantial (κ > 0.7) when used by surgeons who are regularly involved in treating acetabular fractures^2,7^. These differences indicate the steep learning curve to classify this fracture. Therefore, if an algorithm can improve the accuracy and reliability of fracture classification, it will be helpful for resident training.

**Fig. 1:**
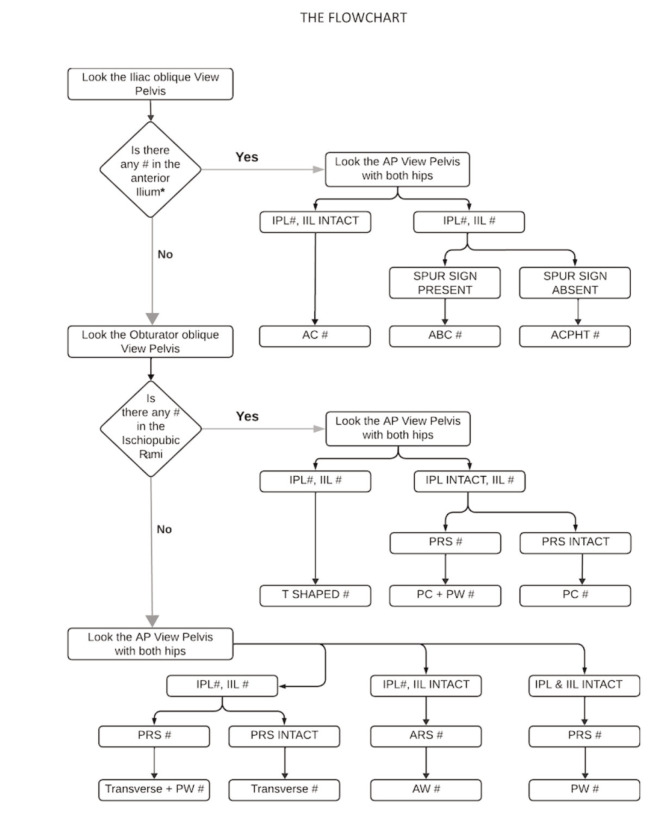
Algorithm for acetabular fracture classification. (IPL: Ilio-pectineal line, IIL: Ilioischial line, ARS: Anterior Rim Shadow, PRS: Posterior Rim Shadow, AC: Anterior column, ABC: Associated both column, ACPHT: Anterior column ± Posterior hemitransverse, PC: Posterior column, PW: Posterior wall, AW: Anterior wall, #: Fracture).

**Fig. 2:**
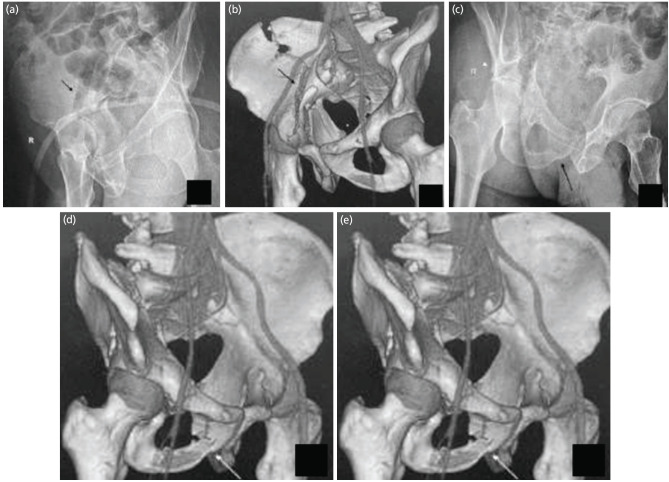
Judet views and 3D CT scan of a patient with acetabular fracture. (a) The Iliac oblique view showing the fracture of the iliac wing (Arrow). (b) The 3D CT showing the fracture of the iliac wing. (c and d): The obturator oblique view and 3D CT showing fracture of the obturator ring (arrows) and absence of spur sign (white arrowhead). (e) The AP view of pelvis with both hips showing the discontinuity of both ilioischial and iliopectineal lines. So according to the flowchart, as there is a fracture of the iliac wing, disruption of both the IL and IP lines and absence of spur sign, it is an anterior column with posterior hemitransverse fracture.

**Fig. 3:**
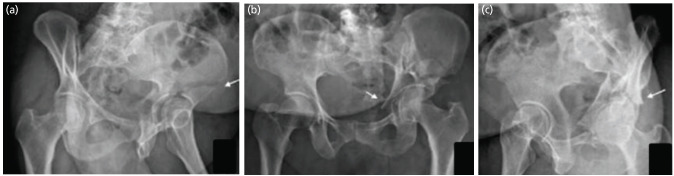
Judet views of a patient with acetabular fracture. (a) Iliac oblique view showing the fracture of the iliac wing (arrow). (b) The AP view of pelvis with both hips showing the discontinuity of both ilioischial and iliopectineal lines. (c) The obturator oblique view showing presence of spur sign (arrow). So according to the flowchart, as there is a fracture of the iliac wing, disruption of both the IL and IP lines and presence of spur sign, it is an associated both column fracture.

**Fig. 4:**
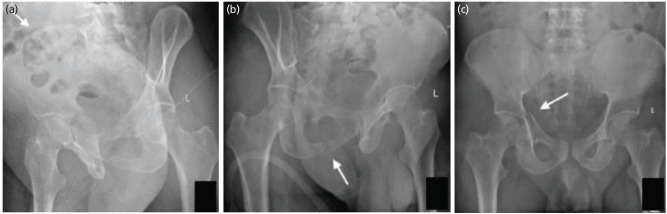
Judet views of a patient with acetabular fracture. (a) Iliac oblique view showing intact iliac wing (arrow). (b) The obturator oblique view showing fracture of the obturator ring (arrow). (c) The AP view of pelvis with both hips showing the discontinuity of both ilioischial and iliopectineal lines (white arrow) and a posterior wall fracture (black arrow). So according to the flowchart, as there is a no fracture of the iliac wing, with fracture of obturator ring and both the IL and IP lines, it is a T type fracture.

**Fig. 5:**
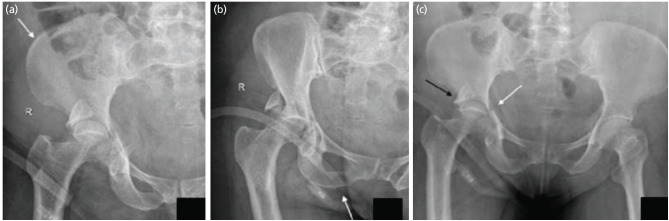
Judet views of a patient with acetabular fracture. (a) Iliac oblique view showing intact iliac wing (arrow). (b) The obturator oblique view showing intact obturator ring (arrow). (c) The AP view of pelvis with both hips showing the discontinuity of both ilioischial and iliopectineal lines (white arrow) and a posterior wall fracture (black arrow). So according to the flowchart, as there is a no fracture of the iliac wing and obturator ring, with disruption of both the IL and IP lines and presence of posterior wall fracture, it is a transverse with posterior wall fracture.

We have devised a new algorithm to classify acetabular fracture as per the Judet-Letournel classification (Fig. 1). In first step of our algorithm, we rule out any iliac wing fracture to eliminate the fracture subtypes having an anterior column component (Fig. 2 and Fig. 3). In the next step, ruling out an obturator ring fracture eliminates fractures with posterior column components and T-type fractures (Fig. 4). Finally, the iliopectineal (IP) line, ilioischial (IL) line, anterior rim shadow, and posterior rim shadow are screened to reach a diagnosis (Fig. 5).

The purpose of the current study was to test if the accuracy and reliability of fracture classification increase using our novel algorithm among resident doctors. We hypothesise that our algorithm helps resident doctors understand the complex anatomy of acetabulum fractures and classify them according to the Judet-Letournel classification.

## Materials and Methods

The current study was a multicentric survey to validate our algorithm. Radiographs of 62 consecutive skeletally mature patients with acetabular fractures operated in our institute and whose CT scans and radiographs (anteroposterior, iliac, and obturator oblique views) were available in our hospital database were selected. Two authors (GJ and GV) with considerable experience in acetabular fracture management independently screened and classified these fractures using radiographs and CT scans. Forty patients, where there was consensus regarding the diagnosis between the two authors and parity between the radiographs, CT scans (including 3D-CT scans), and intra-operative findings, were included in our study. Forty sets of radiographs, each containing anteroposterior, iliac, and obturator oblique views of one patient, were prepared after removing all the patient-related information. From these forty sets, the first twenty were selected as per the date of patient registration and labelled Collection 1, while the subsequent twenty sets of radiographs were labelled Collection 2 (Table I).

**Table I:** Distribution of fractures in both collections.

Sl no.	Fracture type	Collection 1	Collection 2
1	ACPHT	2	1
2	Anterior wall fracture	1	1
3	Associated both column fracture	3	3
4	Posterior column fracture	1	0
5	Posterior wall fracture	4	3
6	T shaped fracture	2	3
7	Transverse with post wall fracture	3	6
8	Transverse fracture	4	0
9	Posterior Column with post. Wall fracture	0	2
10	Anterior column fracture	0	0

ACPHT: Anterior column with posterior hemitransverse fracture

**Fig. 6:**
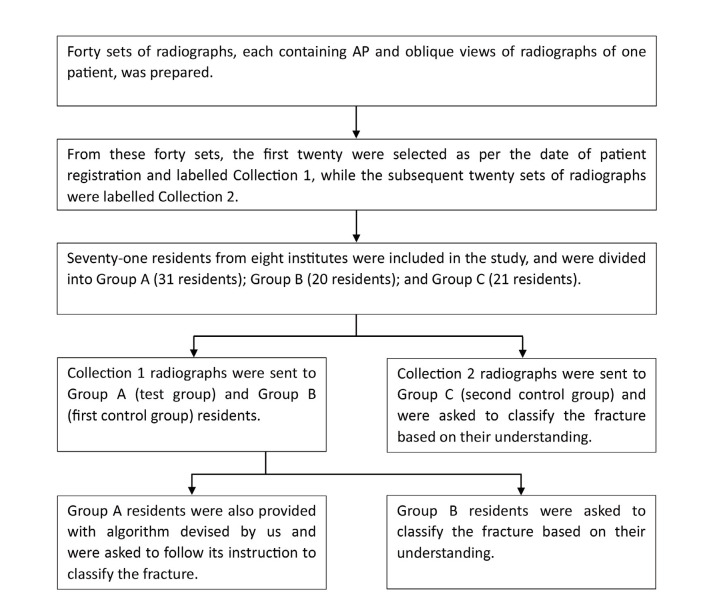
Flowchart depicting the process of the survey.

Resident doctors with 2 to 4 years of training experience (PGY 3 and PGY 4) from eight institutes, which were tertiary care centres with high-volume trauma and acetabular fracture management, were included in the study. Seventy-two out of the 110 eligible residents in these eight institutes accepted our request to participate in our survey. The first thirty-one residents were included in the test group (Group A). Subsequent twenty were included in the first control group (Group B), and the remaining 21 were included in the second control group (Group C). The residents of group A were provided with twenty sets of radiographs included in Collection-1 along with our algorithm and were asked to classify the fractures following the instructions in the flowchart. The residents of Group B were provided with twenty sets of radiographs included in Collection 1, and the residents of Group C were provided with those in Collection 2 and were asked to classify the acetabular fracture based on their own understanding (Fig. 6).

None of the residents were part of the team that selected the radiographs for review. The residents could skip an answer if they were unsure regarding the classification. To calculate the intra-observer reliability, five residents of group A were sent the same sets of radiographs of Collection 1 after an interval of six months and were asked to submit the classification using the algorithm again.

Statistical Analysis: SPSS software was used to analyse the data obtained. Continuous variables were expressed in mean ± standard deviation, and categorical variables were expressed as frequency and percentage. The Chi-square or Fisher's exact test was used to compare categorical data. An Independent t-test or analysis of variance was used to probe quantitative data. The interobserver reliability of the classification with or without the algorithm was assessed by estimating Fleiss kappa. Krippendorff's alpha was estimated to see the reliability of getting a correct answer with or without a flowchart. Intra-observer reliability was estimated using Cohen's kappa. The agreement was interpreted to be poor, slight, fair, good, or excellent according to the criteria suggested by Landis *et al*^8^*.* A P-value less than 0.05 was considered statistically significant.

## Results

The average experience of 31 residents of Group A was 29.87 ± 10.16 months, while that of Group B was 29.83 ± 6.2 months and that of Group C was 28.5 ± 5.4 months (P value-0.81). A total of 1411 unique responses were made during the survey out of which 602 were made by the group A residents (response rate- 97%), 392 by group B residents (response rate- 98%) and 417 by group C residents (response rate-99.3%). The accuracy of group A residents was 53.8%, while that of group B and C was 34.9% and 28.3%, respectively. According to fracture types, compared with both group B and C residents, all fracture types were identified more correctly by the group A residents and the difference was statistically significantly for most fracture types (Table II). The interobserver reliability for fracture classification with an algorithm was fair, while it was slight without an algorithm (Table III). The interobserver agreement to get a correct answer, estimated according to Krippendorf alpha, with algorithm was 0.3013 ± 0.29 and without algorithm (Group B) was 0.152 ± 0.14. The mean intra-observer reliability among five observers was 0.43 (0.13) (Table IV). All residents of group A who were provided with the algorithm, felt the latter to be simple to comprehend and useful.

**Table II:** Accuracy of fracture classification in all three groups and their comparison.

Fracture Type	Group A			Group B			Group C			Group A vs B	Group A vs C
	Correct	Incorrect	UA	Correct	Incorrect	UA	Correct	Incorrect	UA	P value	P value
Overall	324	278	18	137	255	8	118	299	3	0.0001	0.0001
ACPHT	26	32	4	4	34	2	1	20	0	0.0003	0.0009
Anterior wall fracture	7	22	2	3	17	0	3	18	0	0.4959	0.488
Associated both column fracture	46	47	0	16	44	0	17	46	0	0.005	0.005
Posterior column fracture	12	18	1	10	9	1	0	0	0	0.3864	-
Posterior wall fracture	100	23	1	51	28	1	42	20	1	0.0075	0.04
T shaped fracture	41	18	3	13	25	2	15	47	1	0.0006	0.0001
Transverse + posterior wall fracture	35	56	2	26	33	1	31	115	1	0.4947	0.004
Transverse fracture	57	62	5	14	65	1	0	0	0	0.0001	-
Posterior column + Posterior wall fracture	0	0	0	0	0	0	9	33	0	-	-

ACPHT: anterior column with posterior hemitranverse fracture, UA: unattempted

**Table III:** Reliability of fracture classification in both groups assessed by Fleiss kappa.

Fracture Type	Group A	Group B	Group C
Overall	0.318	0.144	0.104
Anterior column + Posterior hemitransverse fracture	0.05	0.001	0.028
Anterior column fracture	0.06	0.132	0.032
Anterior wall fracture	0.088	0.045	-0.002
Associated both column fracture	0.491	0.11	0.079
Posterior column + Posterior wall fracture	0.118	0.2	0.014
Posterior column fracture	0.127	0.004	0.091
Posterior wall fracture	0.707	0.452	0.46
T shaped fracture	0.065	0.037	0.1
Transverse + posterior wall fracture	0.127	0.064	0.117
Transverse fracture	0.191	0.052	0.09

**Table IV:** Intra-observer Reliability estimated in five residents.

	kappa	SE of kappa
Resident 1	0.366	0.118
Resident 2	0.45	0.132
Resident 3	0.427	0.116
Resident 4	0.39	0.12
Resident 5	0.529	0.121
Mean	0.4324	0.1318

## Discussion

The current study showed that our algorithm considerably improved the proficiency of PGY 3 and 4 resident doctors in classifying the acetabular fracture according to the Judet-Letournel classification. The accuracy of diagnosis improved significantly with the algorithm for most fracture types. Furthermore, as evaluated by Fleiss Kappa and Krippendorff's Alpha, the reliability of acetabular fracture classification enhanced from slight to fair with the algorithm. Also, the intra-observer reliability with the algorithm was moderate. Thus, the results support our hypothesis and suggest that screening radiographs for specific radiological signs in a particular sequence to reach a diagnosis is helpful for resident doctors.

In our study, the accuracy of making a correct diagnosis increased by 35% with the algorithm. Most similar previous studies involving resident doctors have also found comparable improvements in accuracy with the help of an algorithm. Saath *et al* and Riouallon *et al*, testing their algorithm, have found that the accuracy among resident doctors increases by 38.5% and 36.7%, respectively, to more than 70%^9,10^. The accuracy of residents included in our study was 54% with the algorithm, which is relatively less than in previous studies. The latter can be explained by the fact that the residents in previous surveys were comparatively senior. The average experience of residents in our study was only 30 months, while in other studies, residents in fourth and fifth years of training were included. Our purpose for enrolling junior residents in the survey was to know the role of an algorithm in early training days, as with time, surgeons can analyse its type even without an algorithm. For instance, Ly *et al* and Prevezas *et al*, surveying senior residents and experienced acetabular surgeons, respectively, reported an accuracy level of more than 50% without an algorithm^11,12^.

The intra-observer reliability estimated in our study for acetabular fracture classification with the algorithm ranged between 0.37 and 0.53 (mean κ = 0.43). Keltz *et al* and Visutipol *et al*, surveying orthopaedic trauma surgeons, have reported a moderate intra-observer agreement in acetabular fracture classification with CT scans and radiographs, respectively^13,14^. Polesello *et al* assessed the intra-observer reliability of acetabular fracture classification among first-year junior resident orthopaedic surgeons and found a fair agreement (κ = 0.29)^15^. Considering the limited experience of our residents, the complex nature of acetabular fractures, and the use of radiographs in our survey, we believe achieving moderate intra-observer reliability shows the usefulness of our algorithm for resident surgeons in classifying acetabular fractures.

Our algorithm is very similar to that proposed by Durkee *et al* concerning the fact that both algorithms consider the integrity of IP and IL lines at the end of the flowchart just before the fracture classification^16^. This approach is distinct from most other algorithms, where the IP and IL lines are evaluated first. Our strategy is advantageous, as studying the integrity of IP and IL lines at the end makes the algorithm comparatively more symmetrical and less complicated. Furthermore, considering IP and IL lines at the beginning is less helpful, as except for posterior wall fracture, all other fractures have either of the lines disrupted. Therefore, almost all fracture types remain unclassified after the first step of the algorithm.

Brandser *et al* suggested six relevant screening areas, including the ilioischial line, iliopectineal line, iliac wing, obturator ring, and posterior wall^17^. However, with their approach, it is not be possible to distinguish between associated-both-column and anterior-column posterior hemi-transverse acetabular fractures. Prevezas *et al* have classified acetabular fractures into three groups based on the integrity of ilioischial and iliopectineal lines^12^. Their algorithm only divides the fracture into groups, and to reach a particular fracture, one has to follow further instructions mentioned in the text.

Many authors, like Shaath *et al*, have advised a similar algorithm based on CT rather than plain radiography^9^. Most previous authors analysing the reliability of 3D- CT in acetabular fractures have found it very reliable^18,19^. Some have even advised not to perform plain radiography routinely to reduce radiation exposure and the cost of treatment^18,19^. However, training resident doctors in analysing plain radiographs is of utmost importance as learning the interpretation of the intra-operative fluoroscopic images are essential in reduction and fixation these fractures^4^.

The strengths of the current study are the multicentric nature of the survey. Also, our algorithm's simplicity and comprehensiveness and the ability to use in plain radiographs is a major advantage. Our algorithm will complement the traditional teaching, which involves learning about anatomy, fracture patterns and approaches. Our algorithm not only helps the resident doctors correctly classify acetabular fractures but also helps them learn various radiographic signs critical for classification. Among the limitations of our study is the inclusion of resident doctors with up to four years of experience; thus, the relevance of our algorithm among experienced senior surgeons needs to be evaluated in future studies. Second, as we have selected radiographs of consecutive patients presented to our institute, certain fracture types were minimally represented in our study. Furthermore, we have not evaluated the results based on the institute to which the residents belong. As residents of eight institutes were involved, dividing the data on that basis was impractical. Therefore, the difference in training of included residents might have affected the outcome.

## Conclusion

From the above study, we can conclude that our algorithm improves the accuracy and interobserver and intra-observer reliability for classifying acetabular fractures according to the Judet-Letournel classification for resident doctors with two to four years of experience.
